# Suitability Evaluation of Crop Variety via Graph Neural Network

**DOI:** 10.1155/2022/5614974

**Published:** 2022-08-09

**Authors:** Qiusi Zhang, Bo Li, Yong Zhang, Shufeng Wang

**Affiliations:** ^1^Information Technology Research Center, Beijing Academy of Agriculture and Forestry Sciences, Beijing 100097, China; ^2^National Engineering Research Center for Agroecological Big Data Analysis & Application, School of Electronics and Information Engineering, Anhui University, Hefei 230601, China; ^3^Beijing Key Laboratory of Multimedia and Intelligent Software Technology, Beijing Institute of Artificial Intelligence, Department of Information Science, Beijing University of Technology, Beijing 100124, China

## Abstract

With the continuous growth of the global population, insufficient food production has become an urgent problem to be solved in most countries. At present, using artificial intelligence technology to improve suitability between land and crop varieties to increase crop yields has become a consensus among agricultural researchers. However, there are still many problems in existing works, such as limited crop phenotypic data and the poor performance of artificial intelligence models. In this regard, we take maize as an example to collect a large amount of environmental climate and crop phenotypic traits data at multiple experimental sites and construct an extensive dataset. Then, we introduce a graph neural network model to learn crop suitability evaluation and finally achieve a good evaluation effect. The evaluation results of the model can not only provide a reference for expert evaluation but also judge the suitability of the variety to other test trial sites according to the data of the current one, so as to guide future breeding experiments.

## 1. Introduction

Crop variety suitability evaluation refers to the suitability of crop variety growth for corresponding planting land. Soil conditions and climatic environments vary significantlyfrom place to place, and the suitability of different crop varieties differs greatly. Select suitable varieties for planting, and then maximize the use of limited land resources to produce more food. Affected by many factors such as the outbreak of new coronavirus pneumonia, climate change, and frequent natural disasters, the world food security situation has become more severe in recent years, which may lead to a further increase in the global hunger population. In this regard, the world food security situation has become more severe in recent years, leading to a further increase in the global hunger population, so that future crop varieties can be accurately planted on suitable land, to improve food production.

Climate change will continue to affect the whole period of crop growth, which has a great impact on the suitability evaluation of crop varieties. Long-term climate change leads to large-scale reallocation of freshwater resources resulting in changes in crop breeding [1, 2]. Literature [3] points out that, due to climate change in the next few years, the total output of crops will decline, which is in great contradiction with the growing food demand of the global population. To alleviate this contradiction, we need to actively explore the relationship between climate change and crop variety adaptability and optimize the utilization of land resources.

Crop phenotypic traits are the intuitive expression of the suitability between crop growth and current land, and the result of the interaction between environmental factors such as soil and climate and crop varieties. Crop variety selection based on crop phenotype was relatively systematic long before technologies such as DNA and molecular markers emerged. Even the same crops and genes will produce different phenotypes in different environments. Ultimately, crop harvest is phenotypic data, not genome. Therefore, direct research and analysis of crop phenotype are the most natural and effective method. However, the biggest problem is that phenotypic data is not enough to support extensive data analysis.

Crop suitability evaluation has always been a major problem in agricultural production, but the currently used evaluation and analysis methods are outdated and have low evaluation accuracy. Most of the existing methods are based on traditional machine learning methods. This method treats each piece of data as an independent sample and lacks the exploration of the relationship between the data. The later introduction of deep learning made the model more powerful in nonlinear fitting but still failed to model higher-order correlations between data.

Given the the lack of variety suitability evaluation dataset, we collected crop variety trait data and environmental-climate data from multiple breeding sites in the past five years (2017–2021), with a total of 10,000 records. Each record includes 15 of trait data and 24 of climate data, and experts are invited to conduct corresponding suitability evaluation, and experts are invited to conduct corresponding suitability evaluations. Considering the high-order complex correlation between crop phenotypic traits and climate data [4–6], we incorporate climate data into the learning suitability assessment. Then, we use the graph neural network to learn the association representation between the data, and finally achieve better evaluation accuracy. Overall, this paper mainly includes the following three contributions:

1. We have collected a large amount of data related to cultivar adaptability, alleviating the difficulty of the scarcity of datasets in the current field.
2. The graph neural network model is introduced into the variety suitability evaluation, and good evaluation results were obtained.
3. The results of the experiments can provide a reference for future breeding programs and improve breeding efficiency.

## 2. Related Works

Variety suitability evaluation is a long-term problem, and many works in this field have guiding significance for agricultural production. Below we briefly introduce some representative works.

### 2.1. Relevant Works of Variety Suitability Evaluation

The authors of [7] believe that environmental climate and genetic factors jointly affect the final yield of crops, so the authors aim to understand the impact of climate on agriculture through methods similar to quantitative genetics, and to improve crop yield through selection, manipulation, and editing of genetic variations. Traditional empirical land assessment and soil surveys rely on expert explanations. They cannot answer future land use issues, such as future climate change, including the availability of water resources, and the introduction of new crop hybrids. In this regard, [8] explores the effect of limited water availability on the growth of various maize hybrids under future climatic conditions. Literature [9] is committed to developing an efficient field high-throughput phenotypic analysis platform to make crop-related data collection more comprehensive and accurate. Literature [10] focuses on the current and long-term needs of society. The authors believe that the future breeding data will integrate genetic, statistical, and gene-phenotypic traits to promote our understanding of functional germplasm diversity and gene-phenotypic-trait relationships in local and transgenic crops. Literature [11] is committed to exploring field climate intelligent crops, using a large amount of data from phenotypic and genomic datasets. The authors integrate genome and crop phenotypic information into specific databases and intelligent platforms and then select the appropriate climate environment to make crops adapt to the environment and ultimately improve crop yield.

### 2.2. Deep Learning in Agriculture

Agriculture is closely related to people's daily life, and its importance at the national level is self-evident. Given the amazing learning ability of deep learning and the rapid accumulation of agricultural data, many researchers have begun to explore how to use the technology to guide agricultural production. Below we briefly introduce some recent works using deep learning for agricultural production and then introduce the application of graph neural networks in agriculture. The impact of weather data on sustainable agricultural production is enormous, but the complex nonlinear relationship between data makes weather data unpredictable. In response,[12] proposes a deep learning predictor with a continuous two-level decomposition structure, which continuously decomposes weather data into four components and then trains a Gated Recurrent Unit (GRU) network as a subpredictor for each component. Literature [13] is dedicated to solving crop management problems in agricultural automation. The authors use convolutional neural network technology to identify weeds in the early stages of crop growth and control the side effects of weeds on crop growth, thereby improving yields. They propose AgroAVNET, a hybrid model based on AlexNet and VGGNET, with a extensive performance improvement compared to existing methods. Literature [14] is dedicated to using past agricultural production data to predict future agricultural production. The authors propose a deep learning model AGR-DL based on CNN and RNN. The experimental results show that the prediction accuracy of the model is better than that of classical algorithms such as SVM, MLP, and AdaBoost. Faced with limited water resources and arable land resources, how to maximize the utilization has become the common goal of researchers. In this regard, [15] proposes an IoT precision agriculture intelligent irrigation system based on deep learning neural network. It can make arable land smarter by using a long short-term memory network to predict the previous day's volumetric soil moisture content and irrigation cycle. The combination of Industry 4.0 and smart agriculture is the future development direction, but IoT devices have always faced the potential risk of being attacked. In this regard, [16] proposes a DDoS attack intrusion detection network based on convolutional neural network, deep neural network, and recurrent neural network, which ensures the security of thousands of IoT-based smart devices.

Literature [17] uses graph convolutional neural networks to encode knowledge implicit in the GO hierarchy. The authors propose a DeepGOA model to predict protein annotations, achieving superior performance to deep learning. Literature [18] is dedicated to exploring the effects of soil composition on vegetation growth, and ultimately to rational irrigation scheduling and optimization of water use tools. The authors construct an end-to-end framework, using graph neural network to learn time graph structure and soil moisture. Literature [19] uses a graph-based recurrent neural network to predict crop yield. The authors further improve the prediction ability of the model by reasonably utilizing the knowledge of geography and time, which is superior to the most advanced methods. Literature [20] is committed to graph neural networks to classify the maturity of avocado. The authors create a set of alligator image data and then use the node classification method of graph neural network to classify them.

The above works have improved the suitability between crops and planting sites. However, there are still many unsolved problems. For example, the dataset collected by [7] is small, and the most important crop phenotypic data in suitability evaluation is only 6 kinds, which is seriously insufficient. In addition, the methods used in most suitability evaluation works are outdated, and there is much room for improvement.

## 3. Data Collection

According to the Bureau of Statistics and China Institute of Commerce and Industry, corn is one of the essential food crops in China, and its crop yield exceeds that of rice and wheat. In 2021, the national grain field was 6.3275 million tons, 1.6 million tons more than the previous year, an increase of 2.6%. Of these, rice production was 21.285 million tons, up 100,000 tons or 0.5% of the prior years; wheat production was 13.695 million tons, up 270,000 tons or 2.0% of the prior years; and and corn production was 27.255 million tons, up 1.64 million tons or 4.6% of the prior year. As of December 2021, China's grain yield was 5805 kg/ha, unchanged from the previous year. Among grain crops, rice yield was the highest at 7,113.4 kg/ha, while corn and wheat yields were 6,291 and 5,863 kg/ha, respectively. Our phenotypic data and climatic data used in this paper are from 14 test trial sites in mainland China, including Beijing-Tianjin-Hebei, Northeast, North China, Huang-Huai-Hai, Northwest, and Southwest. Assessing the suitability of target varieties and planting sites requires large amounts of experimental data, and the corresponding costs are often enormous [21].

### 3.1. Data Introduction

Through the collection and collation of crop experimental data in the past five years, we have 10,000 tabular datasets, each of which describes in detail the multiple traits of a certain maize variety at a certain experimental point, including leaf blight, lodging rate, inversion rate, grey speck disease, plant height, ear height, empty stalk rate, duration period, ear rot, hundred-grain weight, ear length, bald tip length, fresh ear field, acre yield, and relative change of yield. Next, we will detail what each trait dataset means and its possible effect on the crop.

#### 3.1.1. Leaf Blight (LB)

The disease is caused by *Corynespora umbilicus*. It mainly damages leaves, and in severe cases, it also damages leaf sheaths and bracts. It generally starts at the bottom leaf and gradually expands upwards. The disease is widely distributed in all maize-growing regions in the world and generally reduces maize production by 15–20%, and in severe cases, it reduces production by more than 50%. The occurrence and prevalence of the disease are comprehensively affected by many factors such as disease resistance of inbred lines, crop rotation system, climatic conditions, and cultivation measures.

#### 3.1.2. Lodging Rate (LR)

Lodging refers to the phenomenon that crops that grow upright are skewed due to excessive growth or even fall to the ground. Lodging rate refers to the percentage of plants with a slope greater than 45 degrees to the total number of plants. It reflects the tilt or landing of maize plants due to wind and rain or improper management in the growth process of maize. The main reason for corn lodging is the weather, mainly rainy days in the jointing period and storms in the grain-filling period.

#### 3.1.3. Inversion Rate (IR)

It refers to the percentage of plants broken below the ear in the total number of plants after tasseling. This phenomenon generally occurs about ten days before the corn tassel stage, when the corn stalks are easily broken by strong winds. This situation is related to the heredity of varieties and the climatic environment (such as wind speed) of planting sites.

#### 3.1.4. Grey Speck Disease (GSD)

Grey speck disease is one of the most devastating corn diseases in northern China, mainly affecting the leaves. It is mainly harmful to leaves. In the early stages, rounded gray spots without distinct edges form on the surface of the leaves, later turning brown. In severe cases, most of the leaves turn yellow and scorch, the ears droop, the grains are loose and dry, and the 100-grain weight decreases, which seriously affects the yield and quality. The disease is obviously affected by the climate, and it is easy to occur in weather conditions with many rainy days, high air humidity, and poor light.

#### 3.1.5. Plant Height (PH)

Plant height refers to the height of the corn plant. This index has a great influence on the yield and lodging rate of varieties. If the corn plant is too high, it will be more affected by natural disasters such as strong wind and heavy rain during the critical period of corn production. The plant height of corn is greatly affected by fertilization. For example, excessive nitrogen fertilizer but lack of potassium fertilizer will cause the plant to grow too vigorously, and the plant will be too high but the yield will decrease.

#### 3.1.6. Ear Height (EH)

It is the length from the root of the corn to the bottom of the ear of the corn. The lower the ear position of corn is, the stronger the lodging rate is, and on the contrary, lodging occurs easily. Therefore, people prefer the varieties with low ear position and sometimes artificially suppress the ear position. The ear height is mainly determined by the variety but also has a certain relationship with the environment.

#### 3.1.7. Empty Stalk Rate (ESR)

Empty stalk generally refers to corn without ears, and the empty stalk rate generally refers to the percentage of the total number of corn plants without ears or ears without seeds after the corn matures. Empty stalk rate is a common phenomenon in corn production, and the empty bar rate directly affects the level of corn yield. If corn encounters rainy weather during the flowering period, the empty stalk rate of some corn varieties may be as high as 50% to 60%, resulting in a sharp drop in corn yield.

#### 3.1.8. Duration Period (DP)

It refers to the number of days it takes corn to mature from sowing to new seeds. Different varieties of corn have different duration periods, and climatic conditions will also lead to changes in corn duration periods, such as north-south differences. According to the length of the duration period, corn varieties are also divided into early-maturing and late-maturing. Therefore, different regions and different varieties of corn have different duration periods.

#### 3.1.9. Ear Rot (ER)

Corn ear rot is a disease caused by a variety of pathogens, mainly caused by more than 20 kinds of molds such as *Fusarium graminearum*, *Penicillium*, and *Aspergillus*. The disease occurs in all corn-producing regions in China, especially in the rainy and humid southwest. Some pathogenic bacteria that cause this disease, such as *Aspergillus flavus*, can produce toxic metabolites such as aflatoxins, which cause serious harm to the health of humans, livestock, and poultry. The disease is mainly related to the variety, and the humid environment also has a certain influence.

#### 3.1.10. Hundred-Grain Weight (HGW)

Hundred-grain weight refers to the weight of 100 seeds, expressed in grams, and is an indicator of seed size and plumpness. The weight of 100 grains of corn is generally around 26–28 grams. If the variety is good and the planting level is high, it can generally exceed 30 grams. If you want to increase the grain weight, the sowing date can be determined according to the local annual temperature to meet the accumulated temperature demand of the corn, so that the grains are within the suitable grain-filling temperature range. This index is affected by corn size and moisture content and varies by cultivar and growing technique.

#### 3.1.11. Ear Length (EL)

Ear length refers to the length of the whiskers on the tip of the corn cob. It is mainly determined by cultivar genes.

#### 3.1.12. Bald Tip Length (BTL)

Bald tip length refers to the length of the tip and top of the cob when corn is harvested without small kernels. Fresh ear field is determined by various factors such as the quality of corn varieties, soil moisture, soil fertility, pests and diseases, planting density, and planting technology.

#### 3.1.13. Fresh Ear Field (FEF)

Fresh ear field refers to the weight of the mature ear of fresh corn, which has a strong correlation with the yield per mu.

#### 3.1.14. Corn Acre Yield (CAY)

Corn acre yield refers to the weight of dry corn kernels harvested on an acre of land. Differences in geographical environment, varieties, management techniques, etc. may lead to different corn yields.

#### 3.1.15. Relative Change of Yield (RCY)

Relative change of yield refers to the change of corn yield at the planting experimental point relative to the reference group. This index reflects the yield gap between the current experimental variety and the control group and is an important basis for our suitability evaluation.

Considering the impact of environmental and climatic factors on the growth of crops, we also collected daily environmental and climatic data of each experimental point, including temperature, air pressure, and humidity. Then, the climate data of each variety growth cycle were preprocessed: the mean and variance of climate from sowing to maturity of maize varieties were taken, including the maximum temperature (MaxT), average temperature (AT), minimum temperature (MinT), temperature difference (TD), ground pressure (GP), relative humidity (RH), precipitation (P), maximum wind speed (MWS), average wind speed (AWS), wind direction angle (WDA), sunshine time (ST), and wind level (WL). Finally, the above 15 crop phenotypic traits datasets and the climate data of 24 test trial sites were integrated into the variety suitability evaluation data.

### 3.2. Data Preprocess

**Figure 1:**
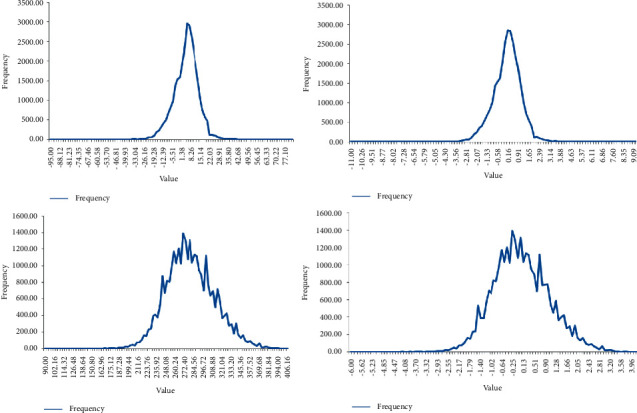
Visualization of numerical distribution of relative change of yield (up) and plant height (down); the left column is the original data, and the right column is after normalization.

We further process the above data so that it can be used for model training. Data processing can be simply divided into two steps: outliers processing and data standardization. Due to environmental differences in different test trial sites, some of the traits are not collected or recorded correctly, resulting in some outliers or missing values in the data. We first manually filter out possible outliers from the data and then fill the average of these feature data. Data standardization is mainly to solve the problem of different dimensions of current data indexes. Different evaluation indexes often have different dimensions and dimension units, and the direct addition cannot correctly reflect the comprehensive results of different index. In order to eliminate the dimensional impact between indexes, data standardization is needed to achieve comparability between datasets. The visualization of data distribution before and after standardization is shown in Figure 1.

In addition, we also carried out data normalization experiments, detailed in Tables 1and 2. The experimental results show that, compared with standardization, normalization reduces the accuracy of the model. We infer that the reason is that the difference between the maximum value and the minimum value in the data of various traits is large, and after normalizing it, the boundaries between many datasets are more blurred, and the model is difficult to identify, so the accuracy of the model decreases.

## 4. Data Correlation Analysis

This chapter is devoted to exploring the relationship between variety suitability and crop traits and the environmental climate data of the test site. To further understand the complex correlations between the datasets, we used the Pearson correlation coefficient to analyze the correlations between the datasets.

There are 39 types of experimental data, including 24 kinds of climate data and 15 kinds of crop traits data. We first analyze the correlation between the datasets, that is, the relationship between the 39 types of data and the proposed label. The recommended variety labels fall into two categories: termination test and continuing test. The former indicates that the crop is unsuitable for the test trial site and should be abandoned. The latter indicates the variety has good performance in the test trial site and could be further tested or planted in large areas. Pearson correlation coefficient is used to measure the correlation between recommended labels and climate and trait data, defined as the quotient of covariance and standard deviation between two variables, as shown in Formula (1). Finally, the relevant conclusions are shown in Table 3. For ease of viewing, we roughen up the data that is more relevant.

(1) $$\begin{array}{c} \gamma =\;\frac{\sum_{i=1}^{n}\left(X_{i}-\bar{X}\right)\left(Y_{i}-\bar{Y}\right)}{\sqrt{\sum_{i=1}^{n}{\left(X_{i}-\bar{X}\right)}^{2}}\sqrt{\sum_{i=1}^{n}{\left(Y_{i}-\bar{Y}\right)}^{2}}}. \end{array}$$

It can be seen from Table 3 that the most relevant data on the recommended label of crop varieties is the relative change of yield, which represents the relative relationship between the current crop yield and the reference group. In addition, the relative humidity, sunshine time, and minimum temperature of the current test trial site environment also have a great impact on variety proposed label.

Among the experts' evaluation criteria of variety adaptability, relative change of yield is the most important reference index, which also conforms to the variety suitability judgment in most cases; that is, yield increase means better adaptability. In other words, the goal of variety suitability can be attributed to increasing crop yield to some extent. It is worth mentioning that, in Section 6.2 of this article, we also conducted experiments that do not use the relative change of yield index to determine the suitability of varieties. Secondly, relative humidity directly reflects the soil moisture status. Relative humidity can increase maize leaf area and yield to some extent [22, 23]. Then, sunshine time directly determines the time of crop photosynthesis, affecting the various stages of crop growth. Maize is a short-day crop, and the whole growth period requires strong light, so sunshine time has a greater impact on crops [24, 25]. Finally, because maize is a light-loving crop, it needs higher temperature during the whole growth period, so the effect of minimum temperature on maize growth is more obvious. If the temperature of corn seedling stage is too low, it will lead to delayed emergence and increased chance of infection. Low temperature during the growth period of maize will lead to dwarfing of plants and poor growth and leaf development. Low temperatures during the ripening period will delay the time for corn to ripen. Literature [26] reaches similar conclusions on the relationship between the minimum temperature and crop growth.

## 5. Graph Neural Network Model for Suitability Evaluation

We treat breed suitability evaluation as a classification task. Unlike previous methods based on machine learning and multilayer perceptual networks, graph neural networks can exploit the correlation between graph datasets to inform suitability evaluation. The task of variety suitability evaluation is to judge the suitability of crops and test trial sites through phenotypic data of crops and climate and environmental data of test trial sites. The input to the model is tabular data, and the final classification result is output. Machine learning or multilayer perceptron methods are generally not suitable for tabular data, and they cannot find optimal solutions to tabular decision manifolds due to lack of proper inductive bias. Second, NLP-based methods are difficult to apply due to the lack of strong semantic associations between columns. In contrast, graph neural networks can model correlations between datasets, using associations to classify tabular data. Furthermore, considering the large differences in the distribution of climate and soil conditions among our test trial sites, the introduction of graph neural networks can also effectively exploit the geographic relationship between test trial sites. When the model is predicting one of the test trial sites, the characteristics of the adjacent test trial sites can be combined with its own characteristics to improve the prediction ability. Next, we briefly introduce the development process of graph neural network, then describe the construction method of graph, and finally compare and analyze the experimental results of the model.

Graph neural network is a new type of neural network. The neural network adopts the idea of bionics to realize modeling by simulating the structure and function of the biological neural network. It can be regarded as a black box where we input specific data features and obtain specific output. Neural network can often learn the mapping relationship between input and output through internal iterations to meet our task requirements. Specifically, classical neural network can be divided into input layer, intermediate layer (also known as hidden layer), and input layer. The number of nodes in the input layer and output layer is often fixed, and the middle layer can be freely specified to hide any number of nodes. Experience shows that the two-layer neural network can approximate any continuous function and has very good data fitting ability.

Graph neural network (GNN) refers to the use of neural network to learn graph structure data and extract and explore the characteristics and patterns in graph structure data. GNN formulates certain strategies for nodes and edges in the graph, converts the graph structure data into standardized representation, and inputs them into various neural networks for node classification, edge information dissemination, graph clustering, and other tasks. Literature [27] proposes to apply convolution operation to graph and proposes graph convolution network (GCN) by clever transformation of convolution operator. The core idea of graph convolution is to learn a function *f* to generate the representation of node *V*_*i*_ by aggregating its own feature *X*_*i*_ and neighbor feature *X*_*j*_, where *j*  ∈ *N*(*V*_*i*_), and N(*V*_*i*_) represents the neighboring nodes near *V*_*i*_. A general graph convolution structure can be represented as shown in Formula (2), which consists of 2 basic operations, aggregation and update, and corresponding weights.

(2) $$\begin{array}{c} X_{\left(l+1\right)}=\text{Update}\left(\text{Aggregate}\left(X_{l},\;W_{l}^{\text{agg}}\right),\;W_{l}^{\text{update}}\right). \end{array}$$

**Figure 2:**
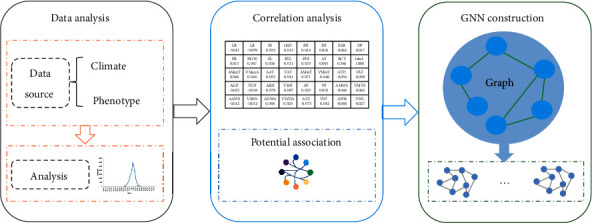
Overall flowchart of the project. The whole project flowchart can be divided into 3 parts: data analysis, correlation analysis, and construction of graph structure. The data analysis part shows the source and numerical distribution of the data; the correlation analysis part gives the relationship between the suitability evaluation indicators and the climate, environment, and crop phenotype data; the graph construction part uses each piece of data as a node to construct a graph and input it into GNN.

The first step in using a graph neural network is to build the graph structure. Firstly, we input all the data with dimension [10000, 39] into the graph structure. Each dataset is regarded as a node, and the distance between nodes is regarded as an edge of the graph. More specifically, we take the chord distance of node characteristics as the edge of the graph network and construct the graph according to the corresponding source node and target node. Secondly, we use a certain number of nodes as losses to train graph networks to meet our performance requirements. Finally, the model was used to assist experts to determine the suitability of varieties and test trial sites. The whole project process is shown in Figure 2.

**Figure 3:**
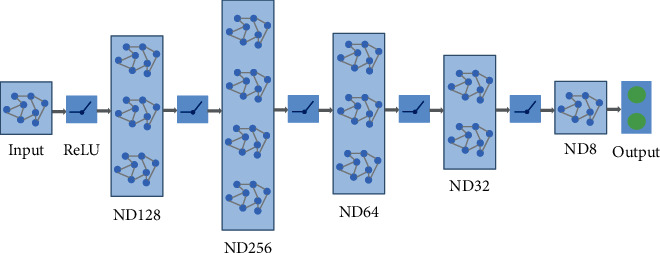
Graph neural network framework. The network consists of an input layer, 4 hidden layers, and an output layer, and the ReLU activation function is used in the middle to increase the nonlinear fitting ability.

The architecture diagram of the graph neural network model is shown in Figure 3. The network loss adopts negative log likelihood loss, which inputs 2 tensors, the prediction tensor and the label. The output of the network obtains the logarithmic probability in the neural network through the log softmax layer, namely, the prediction tensor of the network, and then uses the data label to calculate the loss. In addition, the network uses Adam optimizer [28] to optimize network parameters.

## 6. Experiments

### 6.1. Experimental Results and Analysis

Different from the traditional neural network, the graph network needs to input the entire dataset into the graph at one time and then specify a node as a loss to update the network parameters. Therefore, for a total of 10000 nodes, we choose 50, 100, 400, 700, 1000, and 2000 nodes as losses to update the network, and the results are shown in Table 1.

It can be seen from Table 1 that the prediction performance of the model after data standardization is the best, whether it is the graph convolution network or the traditional machine learning method; that is, the data standardization operation is conducive to improving the prediction accuracy of the model. Then, for the graph neural network, the more the training data are, the more fitting the distribution of the entire data is. In other words, with the increase of the number of training samples, the accuracy of the model is gradually improved.

To verify the performance of the graph neural network model, we conduct comparative experiments using traditional machine learning and neural network methods. We first divide the dataset with data dimension [10000, 39] into training set and test set according to the ratio of 4 : 1, training set: test set = 8000 : 2000. Then, we use traditional neural networks and various machine learning methods for training, including KNN (K-Nearest Neighbor (*N* = 15)), LR (logistic regression), SVM (Support Vector Machine), NB (Naive Bayes classifier), DT (decision tree), RF (Random Forest), MLP (multilayer perceptron), RBFNN (Radial Basis Function Neural Network [29]). Furthermore, we also used a GAT (graph attention neural network [30]) model for comparison.

For a relatively fair comparison, we align the hidden layers of the traditional neural network with the graph neural network. First, we design a six-layer neural network with four hidden layers, the six-layer perceptron. The input feature dimension is 39 and the output feature dimension is 2. Cross entropy is used as loss, probability distribution *p* is expected output, probability distribution *q* is actual output, and cross entropy can be expressed as in Formula (3). For RBFNN and GAT, due to the large difference in network structure, it is difficult to align with GCN, so we choose common network settings. The number of input nodes of GAT is 39, the hidden layer nodes is 64, and the attention head is 2.

(3) $$\begin{array}{c} H\left(p,q\right)=-\sum_{x}\left(p\left(x\right)\text{log}q\left(x\right)\right)+\left(1-p\left(x\right)\right)\text{log}\left(1-q\left(x\right)\right). \end{array}$$

The results obtained by using the above machine learning model for training are shown in Table 2; the higher performance among them is marked in bold. In order to show the performance of the model more comprehensively, we use five indicators for evaluation: accuracy rate, precision rate, recall rate, F1-score, and AUC, and we finally take the average of 20 repeated experiments as the experimental result. Accuracy refers to the ratio of the number of correctly classified samples to the total number of samples, which most directly reflects the performance of the model but is easily affected by class imbalance. The precision rate is the ratio of the number of correctly classified positive examples to the number of classified positive examples, which measures the precision rate of the model. Recall is the ratio of the number of correctly classified positive examples to the actual number of positive examples and measures the recall rate of the model. The F1 score can be regarded as the harmonic average of the model's accuracy and recall, and the calculation formula is as shown in formula (4).

AUC (Area under Curve) is defined as the area enclosed by the coordinate axis under the ROC curve. The closer the AUC to 1.0, the higher the authenticity of the detection method; when it is equal to 0.5, the authenticity is the lowest and has no application value.

(4) $$\begin{array}{c} F_{1}=\;2*\frac{\text{Precision}*\text{Recall}}{\text{Precision}+\text{ Recall}}. \end{array}$$

Among those machine learning methods, random forest, Support Vector Machine, and logistic regression perform the best, while decision tree and naïve Bayesian model perform the worst. Compared with the decision tree, the random forest adopts the integrated algorithm, which is equivalent to integrating multiple decision tree models, and determines the result by voting or averaging each tree, so the accuracy is better than that of the decision tree. In addition,naïveNaive Bayesian model has two basic assumptions. The independent variables are independent of each other, and the continuous independent variables are subject to normal distribution relative to the dependent variables. Combined with the visualization analysis of the numerical distribution of the data in Chapter 3, the independent variable does not fully conform to the normal distribution relative to the dependent variable but fluctuates within a certain range. We believe that this is the main reason for the decline in the accuranaïve the Naive Bayesian model.

We use the 1000 nodes of the GCN model as the training loss accuracy for comparison, which is 74.8%. Compared with traditional machine learning (67.6%), MLP (68.4%), and RBFNN (68.1%), graph neural network achieves higher variety suitability evaluation accuracy with fewer training samples. Furthermore, compared with GAT (73.1%), the GCN model is better in accuracy, but the accuracy is not as good as GAT. Moreover, the GCN model also has a good recall rate, F1, and AUC scores, further verifying the superiority of the model performance.

For the traditional neural network and machine learning algorithms, each variety suitability evaluation dataset is considered as a point feature information, and the algorithm learns the complex mapping relationship between features and labels. In contrast, the graph neural network can transmit information through the graph structure, update the state of hidden nodes through the sum of the weights of adjacent nodes, and effectively utilize the association between feature nodes. For tabular data, different data come from different experimental points, and there are obvious correlations (such as climate factors) between adjacent test trial sites. Therefore, the method of node aggregation can not only mine the similarity between features but also make good use of the association between geographic locations. GAT is generally considered to be an upgrade of GCN. When GAT updates the features of nodes, it first calculates the attention scores of all neighbor nodes and then aggregates the corresponding neighbor features according to the attention scores to better utilize the correlation between features. However, GAT (73.1%) does not perform as well as GCN (74.8%) on our applicability evaluation task. We infer that the reason is that the GAT does not fully utilize the edge information and the network does not learn the connection weights between nodes well.

### 6.2. Further Research

It can be seen from the data correlation in Table 3 that the correlation between the relative change of field index and the suitability evaluation label is much larger than that of other types of data. Therefore, we doubt whether the accuracy of the model is too much affected by the index, resulting in a sharp decline in the performance of the model that is indeed the index, thereby reducing the actual availability of the model. Therefore, we conduct feature data ablation experiments in a targeted manner.

Firstly, the relative changes of yield traits in the overall data were removed, and the other data remained unchanged. Then, 20 groups of experiments were carried out, and the average value was taken as shown in Table 4. The accuracy of the graph neural network model is reduced by about 4%. In contrast, the traditional machine learning and neural network methods decrease greatly, which to some extent shows that the graph neural network learns more data high-order correlation and the model is more robust. In summary, in the absence of relative change of yield index, we can think that the overall performance of the model is within an acceptable range.

## 7. Conclusion and Future Work

With the continuous growth of the world population and the deterioration of the political and commercial situation, food production has become the focus of attention. The use of artificial intelligence technology to improve land suitability and variety adaptability, thereby increasing the yield of food crops, has become the consensus of agricultural researchers. We collected traits and local climate data of 10,000 maize lines in multiple test trial sites, artificial intelligence technology to learn and explore the suitability between maize varieties and test trial sites. Among all artificial intelligence methods, graph neural network has generally achieved good applicability evaluation results, and only 1/10 training samples are used to achieve 75% accuracy.

In the future, we will introduce more factors related to suitability evaluation, such as the genetic sequence of varieties and soil components, and improve the current intelligent technology, so that artificial intelligence can essentially replace expert evaluation. Furthermore, after mastering the data of a variety in a test trial site, the suitability of the variety for other test trial sites can be judged according to the trait data of the variety and the current environmental data. This can eliminate a large number of schemes considered unsuitable by artificial intelligence, thus greatly reducing the cost of trial and error between varieties and test trial sites, accelerating the identification of varieties most suitable for current test trial sites, and ultimately increasing the yield of food crops.

## Figures and Tables

**Table 1:** Accuracy comparison of the following networks with different numbers of training samples.

Number of training samples	50	100	400	700	1000	2000
Raw data	0.651	0.660	0.666	0.674	0.673	0.675
Standardized data	**0.655**	**0.693**	**0.731**	**0.743**	**0.748**	**0.758**
Normalized data	0.551	0.556	0.562	0.555	0.564	0.576

**Table 2:** The performance comparison of traditional machine learning methods and neural network under two data initialization situations. Important data are mar—d in bold.

	KNN	LR	SVM	NB	RF	DT	MLP	RBFNN	GAT	GCN
Accuracy	0.647	0.676	0.671	0.567	0.670	0.553	0.684	0.681	0.731	**0.748**
Precision score	0.628	0.651	0.644	0.562	0.662	0.563	0.670	0.657	**0.743**	**0.687**
Recall score	0.870	0.880	0.892	0.946	0.812	0.583	0.834	0.872	0.708	**0.911**
*F*1-score	0.729	0.748	0.748	0.705	0.730	0.545	0.749	0.750	0.725	**0.783**
AUC score	0.623	0.654	0.647	0.526	0.655	0.483	0.673	0.661	0.732	**0.748**

**Table 3:** Data correlation between 39 types of data and proposed label. The trait data are replaced by abbreviations. Climate data are prefixed with A and V, which represent the mean and variance, respectively.

LB-0.041	LR-0.059	IR-0.052	GSD-0.015	PH 0.014	EH 0.016	ESR-0.062	DP-0.017
ER 0.013	HGW 0.047	EL 0.038	BTL-0.011	FEF 0.033	AY 0.045	**RCY 0.346**	Label 1.000
AMaxT 0.046	VMaxA 0.044	AAT-0.053	VAT-0.041	**AMinT 0.071**	VMinT-0.046	ATD-0.054	VAT -0.058
AGP-0.023	VGP -0.018	**ARH-0.079**	**VRH-0.087**	AP 0.025	VP 0.010	AMWS-0.046	VMVS -0.062
AAWS-0.012	VAWS-0.012	ADWA 0.048	VWDA 0.024	**AST-0.073**	VST-0.042	AWR-0.048	VWL -0.027

**Table 4:** Comparison of the original performance of the model and the performance after removing the relative change of yield (RCY) index.

	KNN	LR	SVM	NB	RF	DT	MLP	RBFNN	GAT	GCN
All data	0.647	0.676	0.671	0.567	0.658	0.553	0.684	0.681	0.731	**0.748**
No RCY	0.588	0.571	0.580	0.552	0.580	0.551	0.615	0.581	0.698	**0.709**

## Data Availability

The data are available from the corresponding author upon request.
